# Mutant Ahi1 Affects Retinal Axon Projection in Zebrafish *via* Toxic Gain of Function

**DOI:** 10.3389/fncel.2019.00081

**Published:** 2019-03-21

**Authors:** Louyin Zhu, Laiqiang Chen, Lingya Yan, Brian D. Perkins, Shihua Li, Baoming Li, Hong A. Xu, Xiao-Jiang Li

**Affiliations:** ^1^School of Life Sciences and Institute of Life Science, Nanchang University, Nanchang, China; ^2^Jiangxi Provincial Collaborative Innovation Center for Cardiovascular, Digestive and Neuropsychiatric Diseases, Nanchang, China; ^3^Department of Human Genetics, Emory University School of Medicine, Atlanta, GA, United States; ^4^Guangdong-Hongkong-Macau Institute of CNS Regeneration (GHMICR), Jinan University, Guangzhou, China; ^5^Department of Molecular Medicine, Cleveland Clinic Lerner College of Medicine of Case Western Reserve University, Cleveland, OH, United States

**Keywords:** axonal decussation, AHI1, CRISPR/Cas9, coiled-coil, gain of function

## Abstract

Joubert syndrome (JBTS) is an inherited autosomal recessive disorder associated with cerebellum and brainstem malformation and can be caused by mutations in the Abelson helper integration site-1 (AHI1) gene. Although AHI1 mutations in humans cause abnormal cerebellar development and impaired axonal decussation in JBTS, these phenotypes are not robust or are absent in various mouse models with Ahi1 mutations. AHI1 contains an N-terminal coiled-coil domain, multiple WD40 repeats, and a C-terminal Src homology 3 (SH3) domain, suggesting that AHI1 functions as a signaling or scaffolding protein. Since most *AHI1* mutations in humans can result in truncated AHI1 proteins lacking WD40 repeats and the SH3 domain, it remains unclear whether mutant AHI1 elicits toxicity *via* a gain-of-function mechanism by the truncated AHI1. Because Ahi1 in zebrafish and humans share a similar N-terminal region with a coiled-coil domain that is absent in mouse Ahi1, we used zebrafish as a model to investigate whether Ahi1 mutations could affect axonal decussation. Using *in situ* hybridization, we found that *ahi1* is highly expressed in zebrafish ocular tissues, especially in retina, allowing us to examine its effect on retinal ganglion cell (RGC) projection and eye morphology. We injected a morpholino to zebrafish embryos, which can generate mutant Ahi1 lacking the intact WD40 repeats, and found RGC axon misprojection and ocular dysplasia in 4 dpf (days post-fertilization) larvae after the injection. However, *ahi1* null zebrafish showed normal RGC axon projection and ocular morphology. We then used CRISPR/Cas9 to generate truncated ahi1 and also found similar defects in the RGC axon projection as seen in those injected with ahi1 morpholino. Thus, the aberrant retinal axon projection in zebrafish is caused by the presence of mutant ahi1 rather than the loss of ahi1, suggesting that mutant Ahi1 may affect axonal decussation *via* toxic gain of function.

## Introduction

Joubert syndrome (JBTS) is a developmental disorder characterized by cerebellar vermis hypoplasia and a midbrain-hindbrain malformation, called the molar tooth sign (Joubert et al., 1969; Louie and Gleeson, 2005). JBTS can be caused by mutations in more than 30 genes, and most of these gene products are important for cilia function. The Abelson helper integration site-1 (AHI1) locus was initially identified as a common helper provirus integration site for murine leukemias and lymphomas (Poirier et al., 1988). Later studies revealed that nonsense or frame-shift mutations in AHI1 are associated with JBTS (Dixon-Salazar et al., 2004; Ferland et al., 2004). Genetic mapping and association studies have also identified *AHI1* as a susceptibility gene for schizophrenia and autism (Levi et al., 2005; Amann-Zalcenstein et al., 2006; Ingason et al., 2007, 2010; Torri et al., 2010). All these findings suggest that Ahi1 is important for early brain development and that its dysfunction is involved in neurological and psychiatric disorders that are closely related to abnormal early development.

Mouse models with Ahi1 mutations have provided very valuable information about the function of Ahi1. Several Ahi1 knockout (KO) mouse models were generated by deletion of exon 2 (Xu et al., 2010), exons 2–5 (Hsiao et al., 2009), or exons 6–7 (Louie et al., 2010). These various mutant mice revealed that Ahi1 is important for early development and neuronal differentiation, as loss of Ahi1 can lead to a smaller cerebellum with an underdeveloped vermis (Lancaster et al., 2011), as well as failure of photoreceptor outer segment formation (Louie et al., 2010; Westfall et al., 2010). However, abnormal axonal decussation, a pathological feature of humans with JBTS (Friede and Boltshauser, 1978; Yachnis and Rorke, 1999), was not seen in Ahi1 mutant mice (Hsiao et al., 2009; Xu et al., 2010; Lancaster et al., 2011).

The lack of abnormal axonal decussation in Ahi1 mutant mice could be accounted for by differences in Ahi1’s gene structures and expression in mice vs. humans. Indeed, the N-terminal region of mouse Ahi1 lacks the coiled-coil domain, which is present in N-terminal Ahi1 in humans and zebrafish. Because over 80% of Ahi1 mutations in humans can yield truncated Ahi1 containing the N-terminal region but lacking the intact W40 repeats and SH3 domain, it is possible that abnormal axonal decussation is caused by the effects of truncated human Ahi1 on axonal projection. In support of this idea, homozygous nonsense mutations of Ahi1 in four families did not result in the JBTS phenotypes (Elsayed et al., 2015). Because of similarities in the N-terminal regions of human and fish Ahi1 proteins, we used zebrafish to examine whether Ahi1 mutations can affect retinal axon projection. Our findings support the notion that mutant Ahi1 lacking the intact WD40 repeats is able to affect axonal projection in the ocular tissues of zebrafish. Our findings also suggest that in human brains, *AHI1* mutations may affect axonal decussation *via* the toxic gain of function by N-terminal AHI1.

## Materials and Methods

### Zebrafish Husbandry

Wild-type AB zebrafish (*Danio rerio*) were maintained and raised at an aquatic habitat recirculating water facility on a 14–10 light-dark cycle. Embryos were hatched in embryo medium E3 solution (5 mM NaCl, 0.17 mM KCl, 0.33 mM CaCl_2_, and 0.33 mM MgSO_4_) and kept at 28.5°C. For suppressing pigmentation, 0.003% PTU (1-phenyl-2-thiourea, Sigma-Aldrich, St. Louis, MO, USA) was added into E3 solution when necessary. Embryos were staged according to somite numbers or hours post-fertilization (hpf). The ethical review committee of Nanchang University approved all experimental procedures. For investigating axonal projection in Ahi1 KO zebrafish, we used the PFA-fixed 4 dpf ahilri^46−/−^ embryos, which were provided by Dr. Brian Perkins and described previously (Lessieur et al., 2017).

### *In situ* Hybridization

cDNA probe for Ahi1 mRNA (exons 3–4) *in situ* hybridization was generated using the following PCR primers: forward (5′-AGTCTCAGGAAATTATCGTGCTT-3′) and reverse (5′-TTTTCCTCTTCCCGCTGGTC-3′). PCR products were purified using a kit (Axygen, AP-PCR-250) and then subcloned into T3 vector (Peasy-T3 Cloning Kit, Transgene). After confirming the DNA sequences by Sangon Biotech, Shanghai, China, the plasmid was digested with NcoI (Takara, 1160A) or SpeI (Takara, 1086A) to linearize cDNA, and then used with an SP6 or a T7 transcription kit to generate DIG-labeled (DIG RNA Labeling Mixture, Roche) anti-sense RNA probes. After purification with a RNA Probe Purification Kit (Omega Bio-Tek, R6248), the probe was diluted with hybridization buffer and stored at −20°C before using.

### Whole-Mount *in situ* Hybridization

The experiment of whole-mount *in situ* hybridization was performed as described by Thisse and Thisse (2008). Fish embryos at different developmental stages were fixed in 4% paraformaldehyde/PBS at 4°C overnight, and then dehydrated in methanol with a gradient from 25 to 100% for storage. When the fixed embryos were analyzed, they were gradiently rehydrated to 0.1% PBST (0.1% Tween-20 in phosphate-buffered saline). Embryos at different developmental stages (24 hpf to 72 hpf) were digested with proteinase K from 1 μg/ml to 80 μg/μl for 6–30 min based on hpf. The treated embryos were then incubated in digoxin-labeled probes for hybridization at 70°C overnight. The embryos were transferred to the preheated 50% SSCT-50% hybridization buffer for 30 min at 70°C, blocked in 10% sheep serum in 0.1% PBST, then incubated with anti-DIG antibody at 4°C overnight. The embryos were then incubated in NBT/BCIP (Sangon Biotech, Shanghai, China) staining solution for visualizing stained signals. Embryos were mounted in methylcellulose and were scanned using a Nikon AZ100 microscope with Nikon Digital Sight DS-Fill digital camera (Nikon, Japan). Images were captured and processed with NIS-Elements F 3.0 (Nikon).

### Morpholino Injection

All morpholinos (MOs) used in this article were obtained from Gene Tools LLC (Philomath, OR, USA). We injected a previously reported ahi1 splice-blocking morpholino, 5′-CCACACTCTGAAAGGGAAAAACATT-3′ (Simms et al., 2012), which is able to target the junction region between intron 12 and exon 13 of zebrafish *ahi1* (ENSDART00000148403.2). The control MO was 5′-CCTCTTACCTCAGTTACAATTTATA-3′. Zebrafish embryo yolks (1–2-cell stage) were microinjected with MO in 1–1.5 nl of distilled water and 0.5% phenol red.

### PCR and DNA Sequencing

For testing morpholino targeting efficiency, we collected 30 WT and *ahi1* MO-injected embryos at 3 dpf and extracted their mRNAs for generating cDNA using Reverse Transcriptase M-MLV (RNase H-; 2641Q, Takara). The knockdown efficiency and specificity of *ahi1* were evaluated by PCR with the primers (forward: 5′-AGATGGGCTGTTTTACTCTC-3′; reverse: 5′-TTCCGCAAGGAGTGAACGTA-3′). The intact *ahi1* and truncated PCR products were separated by gel electrophoresis, and the truncated *ahi1* DNA was amplified by AxyPrep DNA Gel Extraction Kit (AP-GX-250G, Axygen) for sequencing (Sangon Biotech, Shanghai, China).

### CRISPR/Cas9 Targeting

The two sites in the fish *ahi1* gene were selected for CRISPR-Cas9 targeting using Benchling^1^. The first target site is located in the region between 19460–19482 of exon 14 and the second target site in the region between 15102–15124 of exon 11 (ENSDART00000148403). We obtained Cas9 protein and crRNA from Integrated DNA Technologies, Inc. USA (IDT) and used the Alt-R CRISPR-Cas9 System (IDT) to synthesize *ahi1* gRNAs. The gRNAs and Cas9 enzyme were mixed at the final concentration of 20 ng/nl and 200 ng/nl in Nuclease-Free IDTE buffer before injection. The CRISPR/Cas9 gRNA mixture (1–1.5 nl) was injected into embryos at the one-cell stage using a microinjection system (PICOSPRITZER III, Parker, IN, USA). After injection, the injected embryos were collected in E3 solution and incubated at 28.5°C for their development.

We collected uninjected WT and the injected embryos at 3 dpf and extracted the genomic DNA for T7EI mismatch assay using a kit (M0302L, NEB). Two pairs of primers (P2: F, 5′-AAA ACC AGC ACT CAG ATA CAG G-3′, R, 5′-ACT TTA ACG CAT AAC CAT CG-3′; P6: F, 5′-CAT AAA ACT CTT CCG CAC CCT-3′, R, 5′-AGA AGA ATT TTC AAG GCA AGC-3′) were used to amplify the target region. The WT and injected embryo genomic DNAs were amplified using Mastercyler X50i (Eppendorf, USA) with 50 μl PCR reaction. These two PCR products were mixed, and T7 endonuclease I was added into the mixture for incubation at 37°C for 15 min. The mixture was then analyzed by gel electrophoresis.

The PCR-amplified products were also isolated by gel electrophoresis and extracted using an E.Z.N.A.^®^ Gel Extraction Kit (D2500901, Omega Bio-Tek, USA). The PCR products were subcloned into T3 vector (Invitrogen™ TOPO™ TA Cloning™ Kit, 450641, Invitrogen, USA) for DNA sequencing (Macrogen Corp., Rockville, MD, USA).

### Retinal Axon Labeling

Lipophilic dyes were injected separately into the eyes of zebrafish embryos at 4 dpf using a microinjection system with the following dyes: DiI (1,1′-Dioctadecyl-3,3,3′,3′-Tetramethylindocarbocyanine Perchlorate; Molecular Probes, cat.-no. D-282) and DiD (1,1′-Dioctadecyl-3,3,3′,3′-Tetramethylindodicarbocyanine Perchlorate; cat. no. D-307). The injected embryos were then incubated at 4°C overnight to allow the dyes to travel along the lipid membrane of retinal axons. Imaging analysis was done using a confocal microscope (Olympus FV1000, Japan) with Z-stack model to ensure that a maximum of axon projections were completely scanned. The length of each retinal axon was calculated from the retina structure edge to the end of the retinal tract, and the eye size was measured by threshold model of Image J.

### Statistical Analysis

Results are presented as means ± SEM, which were obtained using GraphPad Prism 7 (GraphPad Software, San Diego, CA, USA). Unpaired Student’s *t*-test was used to compare two groups, and one-way ANOVA with a Tukey’s multiple comparison test was used for multiple group comparison. A significant level was considered with *p* values < 0.05.

## Results

### Ahi1 Structure and Expression in Zebrafish

**Figure 1:**
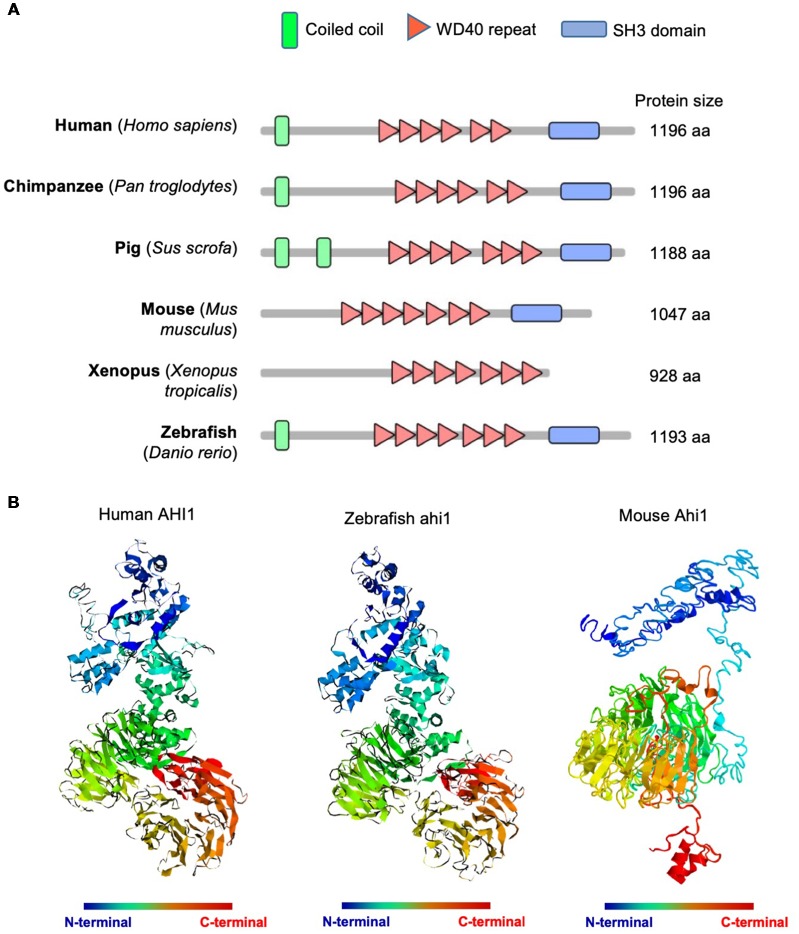
Abelson helper integration Site-1 (AHI1) was highly conserved in multiple animals, from mammals to teleost.** (A)** Schematic diagram of AHI1 proteins in different species. The protein domains were predicted by SMART (http://smart.embl-heidelberg.de/).** (B)** The predicted three-dimensional structures of human, zebrafish, and mouse AHI1 proteins, which were obtained using I-TASSER (https://zhanglab.ccmb.med.umich.edu/I-TASSER/).

Human AHI1 is a cytoplasmic protein containing an N-terminal coiled-coil domain, multiple WD40 repeats, and a C-terminal SH3 domain (Jiang et al., 2002). Its multiple protein-binding domain motifs suggest that AHI1 functions as a signaling or scaffolding protein involved in protein-protein interactions (Jiang et al., 2002; Ferland et al., 2004). AHI1 orthologs are found in all vertebrates and share similar structures of WD40 repeats and the C-terminal SH3 domain (Figure 1A); however, Ahi1 in mouse and *Xenopus* lacks the N-terminal coiled-coil that is present in the primate, pig, and zebrafish (Figure 1A). Using the automated protein structure tool I-TASSER^2^ to analyze three-dimensional structures of human and fish Ahi1, we found that the predicted structures of both human and fish Ahi1 proteins are highly similar (Figure 1B). On the other hand, mouse Ahi1, which lacks an N-terminal coiled-coil domain, shows a three-dimensional structure that is quite different from human and fish Ahi1. These differences suggest that the N-terminal coiled-coil domain may influence the structure and interactions of N-terminal AHI1 with other proteins.

**Figure 2:**
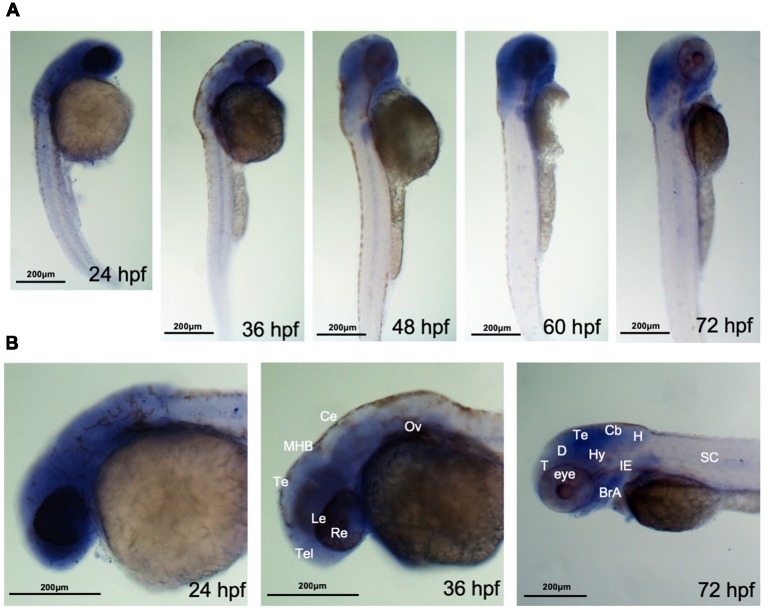
ahi1 is highly expressed in zebrafish cephalosome. **(A)** Whole-mount *in situ* hybridization showed the spatiotemporal expression of *ahi1* mRNA from 24, 36, 48, 60, and 72 hpf. **(B)** At the very early developmental stages (24 hpf and 36 hpf), the rostral feature of *ahi1* mRNA expression revealed that *ahi1* is more highly expressed in whole brain and ocular tissue compared with 72 hpf. T, telencephalon; D, diencephalon; Te, tectum; Hy, hypothalamus; Cb, cerebellum; H, hindbrain; BrA, brachial arches; IE, inner ear; SC, spinal cord.

It has been reported that ahi1 expression in fish is also different from expression in mouse, as mouse Ahi1 is absent in glia and the cerebellum, whereas fish Ahi1 is ubiquitously expressed in the whole body from 2.5 dpf to 5.2 dpf (Doering et al., 2008). However, how ahi1 is expressed in the early developmental stages in zebrafish and whether mutations or loss of ahi1 affects axonal projection during early brain development remain to be investigated. We therefore performed *in situ* hybridization using an antisense oligonucleotide probe to exons 3 and 4 in the fish *ahi1* gene. Whole-mount *in situ* hybridization for *ahi1* mRNA in zebrafish during the early embryonic development stage (from 24 hpf to 72 hpf) demonstrated that *ahi1* is expressed throughout the brain and body (Figure 2A). Importantly, *ahi1* expression in the ocular tissues is abundant at very early developmental stages (24 hpf and 36 hpf) and becomes attenuated at 72 hpf (Figure 2B).

### *Ahi1* Expression and Axonal Projection in Ocular Tissues

**Figure 3:**
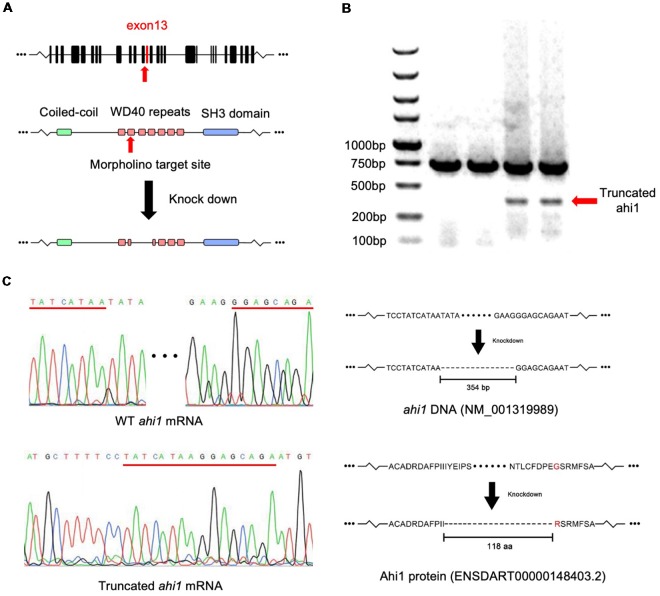
Targeting the zebrafish *ahi1* gene using morpholino. **(A)** The morpholino was designed to target the junction of intron 12 and exon 13 (I12E13) of *ahi1*, which encodes the second WD40 repeat region of the ahi1 protein. The morpholino targeting leads to a deletion 354-bp loss of *ahi1* mRNA, covering almost the whole of exon 12 to exon 13, and results in generation of a mutant ahi1 protein. **(B)** Mutant *ahi1* mRNA was detected by RT-PCR in *ahi1* morpholino-injected zebrafish. **(C)** The sequencing analysis of truncated ahi1 PCR product showed a deletion (354 bp) by morpholino, resulting in the generation of a mutant ahi1 with a 118 amino acid deletion.

Axonal decussations reflect the midline crossing of nerve tracts from one hemisphere of the brain to the contralateral sense organ or limb. In zebrafish embryos, most retinal ganglion cell (RGC) axons project to the contralateral optic tectum. Due to the simplicity and accessibility of the projection of RGC axons to the contralateral optic tectum in zebrafish, this axonal projection has been used to study how axon pathways develop and cross the midline (Hutson and Chien, 2002; Doering et al., 2008; Xu et al., 2010). The enrichment of ahi1 in the eye of early developing fish allowed us to examine whether altering ahi1 expression could affect the RGC axonal projection and eye morphology. To do so, we first knocked down *ahi1* in zebrafish using a morpholino (MO) to target the junction of intron 12 and exon 13 (I12E13) of the fish *ahi1* gene (Simms et al., 2012), which encodes the second WD40 domain in the middle region of fish *ahi1* (Figure 3A). Zebrafish embryos at the 1-cell stage were injected with the *ahi1* MO, and the injected embryos were collected for analysis after 3 dpf. Because of a lack of an antibody to label fish ahi1, we used RT-PCR to detect the expression of *ahi1*. In the *ahi1* MO-injected embryos, RT-PCR with primers to amplify the *ahi1* gene containing exon 11 and 16 clearly demonstrated the presence of a truncated *ahi1* product at approximately a 20% level of the total *ahi1* (Figure 3B). DNA analysis confirmed a deletion of exons 12 and 13, which resulted in a 118 aa deletion and generated a mutant ahi1 form lacking the intact WD40 repeats (Figure 3C).

**Figure 4:**
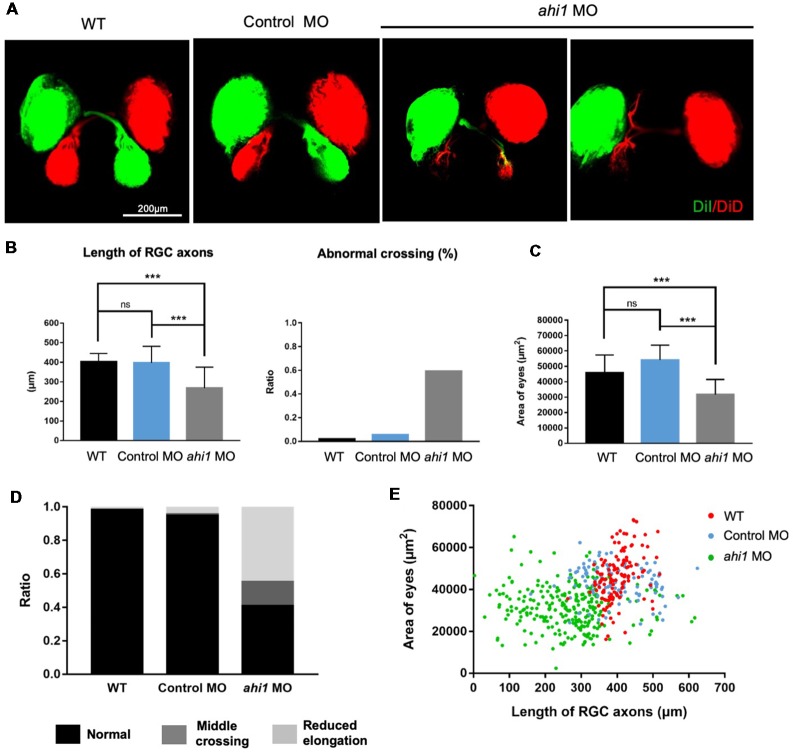
The *ahi1* morpholino-injected embryos exhibited retinal ganglion cell (RGC) axon projection defects at 4 dpf. **(A)** The normal retinotectal projection pattern of WT or control morpholino (Control MO)-injected embryos and defective retinotectal projections in the *ahi1* morpholino (*ahi1* MO)-injected embryos. Scale bar: 200 μm. **(B)** The statistical results of the RGC axon length and abnormal middle crossing of WT (*n* = 106), Control MO- (*n* = 112), and *ahi1* MO- (*n* = 266) injected embryos. **(C)** The statistical results of eye sizes of WT and *ahi1* MO-injected embryos. **(D)** The ratios of embryos with normal retinal axon projection (Normal), abnormal middle crossing (Middle crossing), and reduced axon longation (Reduced elongation) in the WT, control MO-, and *ahi1* MO-injected embryos. **(E)** The two-dimensional scatter diagram for eye size in each group. The X-axis represents the length of OT (μm^2^), and the Y-axis represents the size of eyes (μm^2^). Error bars denote SEM. ***p < 0.0001 was determined *via* Student’s *t*-test.

We then examined the RGC axon projection and eye morphology in *ahi1* MO-injected zebrafish at 4 dpf. The fish eye tissues were fixed and then injected with dye (DiI and DiD) unilaterally to label one side of projected axons and eye tissues, respectively. In this way, the merged images would clearly reveal axonal decussation and eye size. The control eyes of zebrafishes showed symmetric and intact axonal decussation. In the *ahi1* MO-injected fish eyes, failure of the RGC axons to exit the retina was observed frequently. For those eyes with the RGC axon projection, there were reduced or ipsilateral axonal projections, as well as decreased eye sizes (Figure 4A). Quantification of the lengths of nerve projection and 59.25% of fish with abnormal middle crossing verified that *ahi1* MO can disrupt the RGC axon projection (Figure 4B). Furthermore, the area of eyes is also significantly reduced in the *ahi1* MO-injected embryos compared with the control MO group (Figure 4C). The abnormal RGC axon projection was also verified by the ratios of different patterns of axon projection in WT (normal 98.0%; middle crossing 0%, reduced elongation 2.0%), control MO- (normal 94.5%; middle crossing 1%, reduced elongation 4.5%), *ahi1* MO- (normal 41%; middle crossing 14%, reduced elongation 45.0%) injected embryos (Figure 4D) and the two-dimensional scatter diagram for WT, control MO-, or *ahi1* MO-injected embryos (Figure 4E).

**Figure 5:**
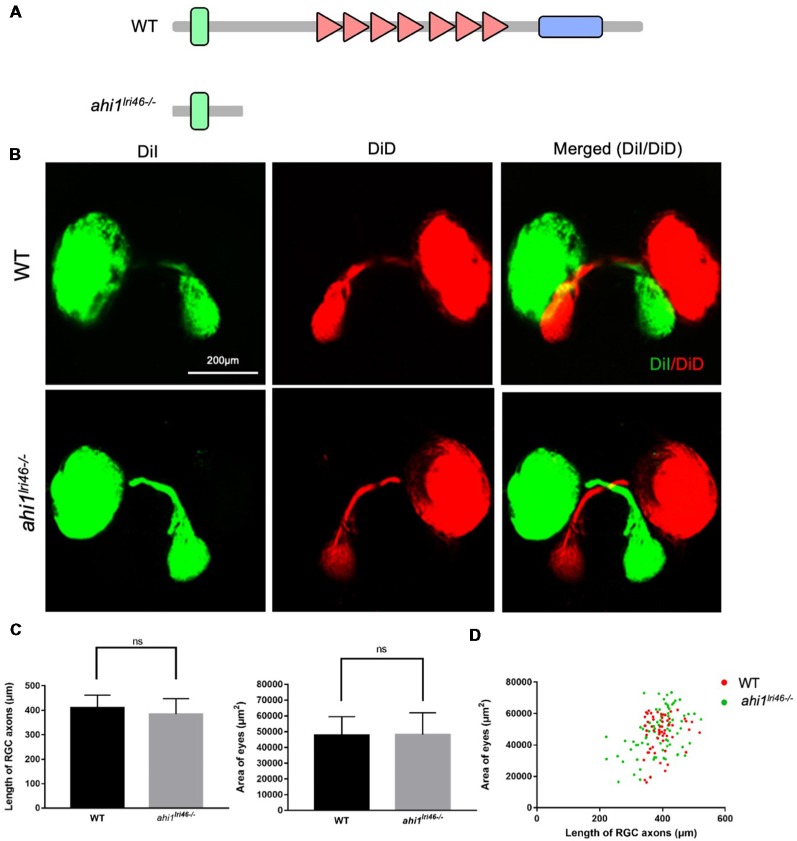
The *ahi1* knockout (KO) line *ahi1lri*^46−/−^ embryos exhibited no significant difference from WT in optic nerve projection length and eyes size. **(A)** The *ahi1lri*^46−/−^ zebrafish has depleted most of the region of the *ahi1* gene and only retains the N-terminal coiled-coil domain. **(B)** Fluorescent images of WT and *ahi1lri*^46−/−^ zebrafish optic nerve projections at 4 dpf. Scale bar: 200 μm. **(C)** The quantified optic nerve projection length and eye sizes of WT and *ahi1lri*^46−/−^ embryos. **(D)** The two-dimensional scatter diagram for the eye size of WT and *ahi1lri*^46−/−^ embryos; the X-axis represents the length of optic nerve projection (μm^2^), and the Y-axis represents the size of eyes (μm^2^). ns, not significant. Error bars denote SEM.

*ahi1* KO fish models are known to cause ciliopathy and abnormal early development (Simms et al., 2012; Elsayed et al., 2015; Lessieur et al., 2017). However, the potential RGC phenotype in the *ahi1* KO models has yet to be investigated. We next examined the RGC axon projection and eye morphology in *ahi1* KO fish. The *ahi1* null (*ahi1lri*^46−/−^) fish model was established by targeting exon 5 of the fish *ahi1* *via* TALENs, which results in a complete loss of the WD40 repeats and SH3 domain of the protein (Lessieur et al., 2017; Figure 5A). Using embryos at 4 dpf, we stained the RGC projection and eyes and found that both *ahi1lri*^46−/−^ and WT embryos showed the same eye sizes and indistinguishable retinotectal projection structure (Figure 5B). We did not find any abnormal middle crossing in *ahi1lri*^46−/−^ fish. Quantitative results also revealed no significant differences in the length and integrity of the RGC axons and size of eyes between these two groups (Figures 5C,D).

### CRISPR/Cas9-Mediated Ahi1 Truncation Affects Axonal Projection

**Figure 6:**
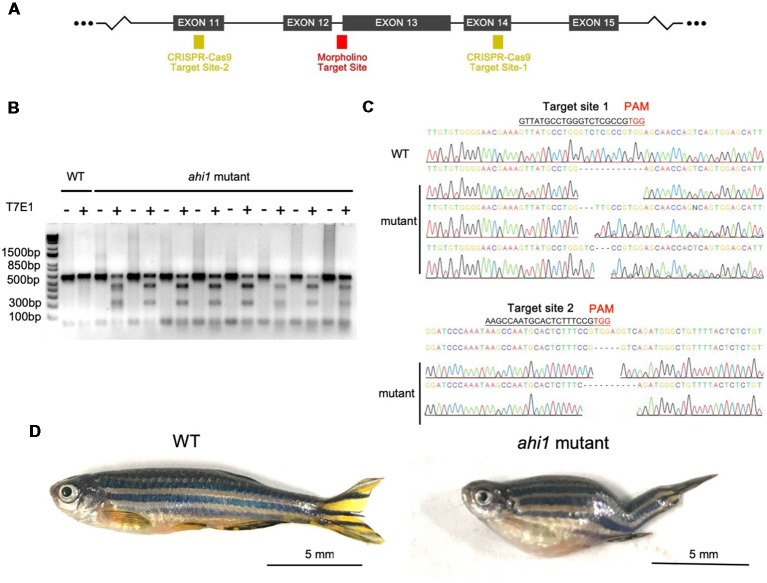
*Ahi1* mutations *via* CRISPR/Cas9 lead to abnormal development of zebrafish. **(A)** The CRISPR/Cas9 target sites were designed to create mutations in exon 12 and exon 14, which were close to the *ahi1* morpholino targeting site. **(B)** T7E1 assay results showed cleaved *ahi1* DNA products by CRISPR/Cas9. **(C)** Sequencing data showing *ahi1* DNA mutations in the *ahi1* CRSIPR/Cas9-targeted (*ahi1* mutant) embryos. **(D)** The morphology of WT and *ahi1* mutant zebrafish at 3 months.

The lack of a defective eye phenotype in *ahi1lri*^46−/−^ fish indicates that this phenotype may be caused by a toxic gain of function of mutant ahi1, rather than the complete loss of ahi1. Given that morpholino can generate truncated mRNA (König et al., 2007; Morcos, 2007), it is possible that mutant ahi1 lacking the intact WD40 repeats generated by morpholino may affect axonal projection in a manner similar to defective axonal decussation in patients caused by *AHI1* mutations that yield truncated AHI1. To validate this idea, we further used CRISPR/Cas9 to generate truncated ahi1 in zebrafish. We designed two gRNAs to target exon 11 and exon 14 in the fish *ahi1* gene, which can create mutations to truncate ahi1 protein in the middle ahi1 region, resulting in truncated ahi1 lacking the intact WD40 repeats and the SH3 domain (Figure 6A). The zebrafish embryos were injected with these gRNAs and Cas9 protein at the one-cell stage. The injected embryos were then collected at 4 dpf after injection for analysis. Because mosaic mutations are usually caused by CRISPR/Cas9, we had to screen and identify those zebrafish that did not develop normally and might contain the targeted ahi1 gene in their ocular tissues. Indeed, in the fish showing abnormal development, T7E1 assays of their eye tissues verified that CRISPR/Cas9 targeting *ahi1* had multiple *ahi1* DNA fragments and reduced levels of intact *ahi1* (Figure 6B). DNA sequencing verified the indel mutations in the targeted site of the fish *ahi1* gene, which could result in truncated ahi1 (Figure 6C). The CRISPR/Cas9-targeted fish showed abnormal size and curved body axis at 3 months compared to wild-type fish (Figure 6D), suggesting that *ahi1* mutations created by CRISPR/Cas9 can affect the normal development of fish.

**Figure 7:**
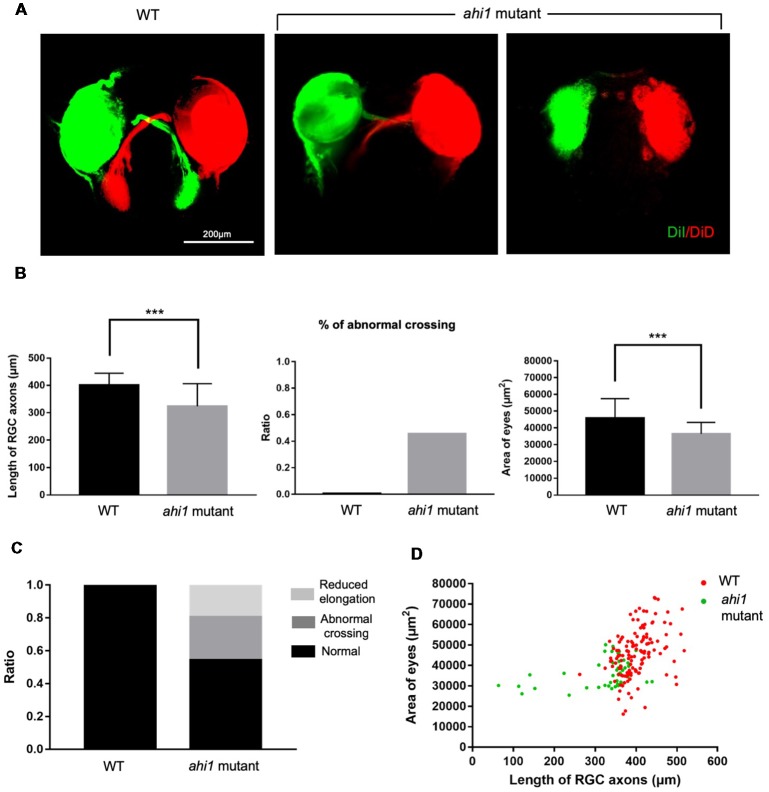
*Ahi1* CRSIPR/Cas9-targeted embryos exhibited abnormal RGC axon projection patterns compared to WT embryos. **(A)** Fluorescent images of the optic nerve projection of WT and *ahi1* CRSIPR/Cas9 targeted (*ahi1* mutant)-embryos at 4 dpf. Scale bar = 200 μm.** (B)** The statistical results of RGC axon length, abnormal crossing, and eye size of WT (*n* = 59) and *ahi1* mutant (*n* = 46) embryos. **(C)** The ratios of embryos with normal retinal axon projection, abnormal middle crossing, and reduced axon elongation in WT and *ahi1* mutant embryos. **(D)** The two-dimensional scatter diagram for the eye area of each WT or *ahi1* mutant embryos. The X-axis represents the length of OT (μm^2^), and the Y-axis represents the size of eyes (μm^2^). Error bars denote SEM. ***p < 0.0001.

Next, we examined eye morphology and axonal projection in the CRISPR/Cas9-targeted fish, as we did previously. We found the same phenotypes (abnormal axonal projections and eye size reduction) in the CRISPR/Cas9-targeted fish embryos at 4 dpf (Figure 7A). In other embryos that did not show *ahi1* mutations, we found no such phenotypes. Quantitative results also showed that CRISPR/Cas9 targeting could yield defective axonal projection phenotypes, including reduced RGC axon projection length, the presence of abnormal crossing, and decreased eye size (Figure 7B). Taken together, using CRISPR/Cas9 to generate truncated ahi1, we also observed similar phenotypes of abnormal axonal projection in the fish ocular tissues. By measuring the ratio of different patterns of axon projection (Figure 7C) and the two-dimensional scatter diagram for WT and CRSIPR/Cas9-mediated *ahi1* knockout (*ahi1* KO) embryos (Figure 7D), we also confirmed abnormal RGC axon projections after targeting the *ahi1* gene by CRISPR/Cas9.

## Discussion

Most *AHI1* mutations in JBTS patients result in truncated proteins lacking the intact WD40 repeats and the SH3 domain. JBTS was therefore thought to be due to loss of AHI1 function. Using zebrafish as a model to target the fish *ahi1* gene, however, we provided the first demonstration that mutant ahi1 lacking the intact WD40 repeats can disturb RGC axon projection, suggesting that *AHI1* mutations can affect neuronal function *via* a toxic gain of function, although its complete loss of function may produce other phenotypes (Elsayed et al., 2015; Lessieur et al., 2017).

Our findings consist of three lines of evidence to support the above. First, morpholino targeting can cause abnormal midline crossing of the retinotectal projection. Second, CRISPR/Cas9 targeting can also replicate this phenotype. Third, the complete loss of *ahi1* in fish failed to yield the phenotype of defective retinotectal projection, ruling out the involvement of loss of function for the defective axonal projection. Because the retinotectal projection represents axonal formation and projection during very early brain development in zebrafish (Stuermer, 1988; Trowe et al., 1996; Picker et al., 1999), the results from our study support the notion that ahi1 is critical for neuronal differentiation and growth during the early development stage in zebrafish and are also consistent with earlier reports that *AHI1* is a susceptibility gene for schizophrenia and autism, which are also abnormal developmental brain disorders (Levi et al., 2005; Amann-Zalcenstein et al., 2006; Ingason et al., 2007, 2010; Torri et al., 2010).

Previous studies have established several *Ahi1* KO mouse models by deleting different exons in the *Ahi1* gene (Hsiao et al., 2009; Louie et al., 2010; Xu et al., 2010). Although these mice may also generate truncated Ahi1, they did not display axonal decussation abnormalities. One possible explanation is that the mouse Ahi1 N-terminal region lacks a coiled-coil domain, which can be a binding site to interact with many other proteins or can facilitate the interactions of other binding domains with partners (Grigoryan and Keating, 2008). Human and zebrafish Ahi1 do contain this coiled-coil motif, which may facilitate the abnormal interactions of the truncated Ahi1 with other proteins to cause a gain of function, even when truncated Ahi1 is expressed at the endogenous level. It seems that zebrafish eye is very sensitive to the toxicity of mutant Ahi1 without the intact WD40 repeats, because the abnormal retinal axon projection occurs when full-length Ahi1 is not completely depleted and when about 20% of the *ahi1* gene was targeted by morpholino to generate mutant ahi1 fish. In humans, although JBST is a recessively inherited developmental brain disorder, heterozygous AHI1 mutations were associated with neurological symptoms (Tory et al., 2007; Otto et al., 2011), supporting a gain of function of AHI1 mutations. In mouse neurons, axonal projection can be affected by a high dose of truncated Ahi1 because overexpressing mouse Ahi1 N-terminal fragments in neuronal cells can also suppress neurite differentiation (Sheng et al., 2008; Weng et al., 2013). The species-dependent sensitivities to mutant AHI1 toxicity could be due to differences in AHI1 sequences, binding partners, and axonal structures in different species.

The toxicity of mutant ahi1 in zebrafish is also supported by previous findings. Truncated fish ahi1 lacking the intact WD40 domains, but not truncated fish ahi1 containing the intact WD40 and SH3 domains, could affect zebrafish development (Elsayed et al., 2015). Thus, a toxic gain of function of AHI1 is likely to be protein context-dependent. Our findings suggest that truncated ahi1 lacking the intact WD40 and SH3 domains can affect axonal projections in the fish eye tissues.

The highly stereotyped retinotectal pathway in zebrafish is determined by complex mechanisms that underlie axonal outgrowth and pathfinding. Axonal outgrowth relies critically on the active intracellular transport that supplies mitochondria, proteins, and other molecules to nerve terminals. Mouse Ahi1 is found to be involved in intracellular trafficking (Sheng et al., 2008; Westfall et al., 2010; Xu et al., 2010), which is consistent with the function of the WD40 and SSH3 domains (Huang et al., 2012; Jain, 2018) and is critical for axonal integrity and projection. Full-length Ahi1 is known to form a stable complex with huntingtin associated protein-1 (HAP1), which is also involved in intracellular trafficking (Sheng et al., 2008; Xiang et al., 2014). Although N-terminal Ahi1 does not bind Hap1 (Weng et al., 2013), an Ahi1 missense mutation was found to inhibit its interaction with Hap1 (Tuz et al., 2013). Also, the toxicity of N-terminal Ahi1 can be attenuated by overexpressed Cend1/BM88, a neuronal protein that mediates neuronal differentiation (Weng et al., 2013). Overexpressed Cend1/BM88 may bind toxic truncated Ahi1 to prevent its abnormal interactions with other proteins and its toxic effects on neuronal differentiation. In addition, truncated Ahi1 could also affect cilia function to impact axonal projection. In combination with these previous discoveries, our current findings from the zebrafish model support the idea for a toxic gain of function of mutant Ahi1, opening up a new avenue to investigate the pathogenesis of diseases associated with AHI1 mutations.

## Author Contributions

HX, SL, and X-JL conceived and designed the experiments. LZ, LC, and LY performed the experiments and analyzed the data. BP provided *ahi1* KO fish embryos. BL provided advice. LZ and X-JL wrote the manuscript. and SL and HX edited the manuscript.

## Conflict of Interest Statement

The authors declare that the research was conducted in the absence of any commercial or financial relationships that could be construed as a potential conflict of interest.

## Footnotes

^1^https://benchling.com/academic

^2^https://zhanglab.ccmb.med.umich.edu/I-TASSER/
